# Supplementary material for: Organizing space through saccades and fixations between primate posterior parietal cortex and hippocampus
Source: Nat Commun. 2024 Dec 1;15:10448. doi: 10.1038/s41467-024-54736-7 (PMC11609276; doi:10.1038/s41467-024-54736-7)
Supplement: Supplementary file 1 — Supplementary Information [file 41467_2024_54736_MOESM1_ESM.pdf]

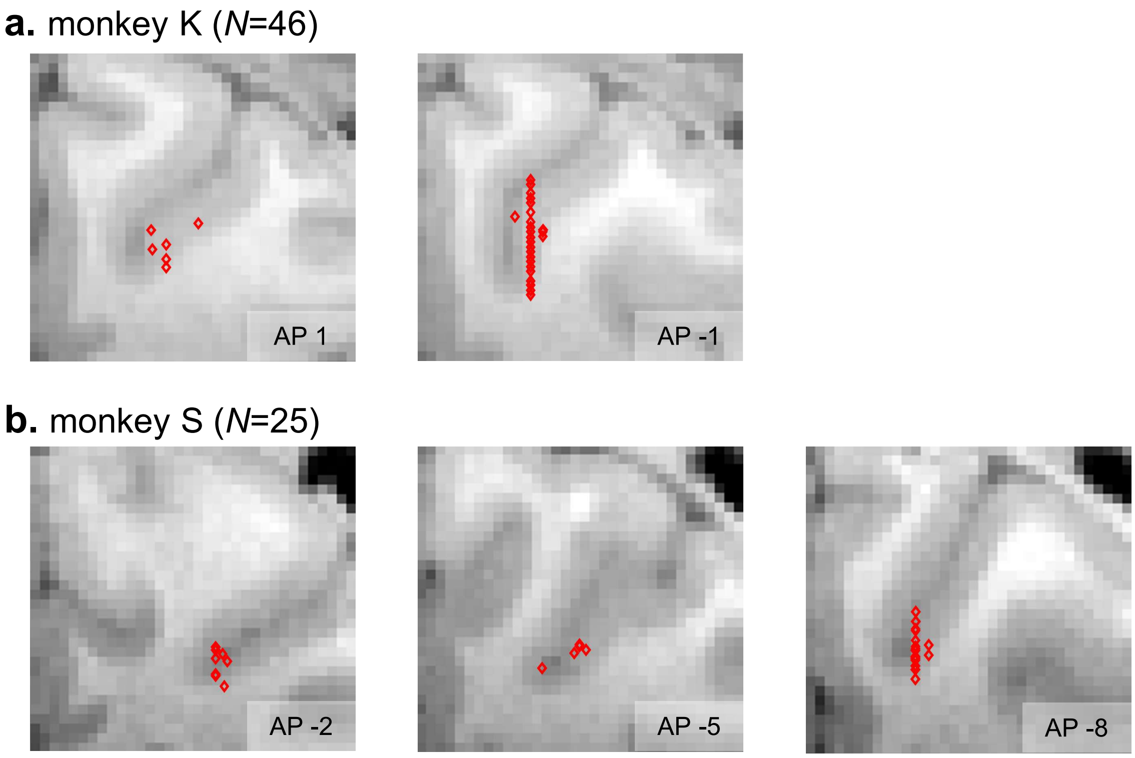

Supplementary Fig. 1 Detailed posterior parietal recording sites. a,b Coronal MRI slices showing the sites where the 111 parietal neurons were recorded, in Monkey K (**a**; $N_{neurons}$ =57, $N_{sessions}$ =46) and Monkey S (**b**; $N_{neurons}$ =54, $N_{sessions}$ =25). 1 pixel corresponds to 1x1mm.

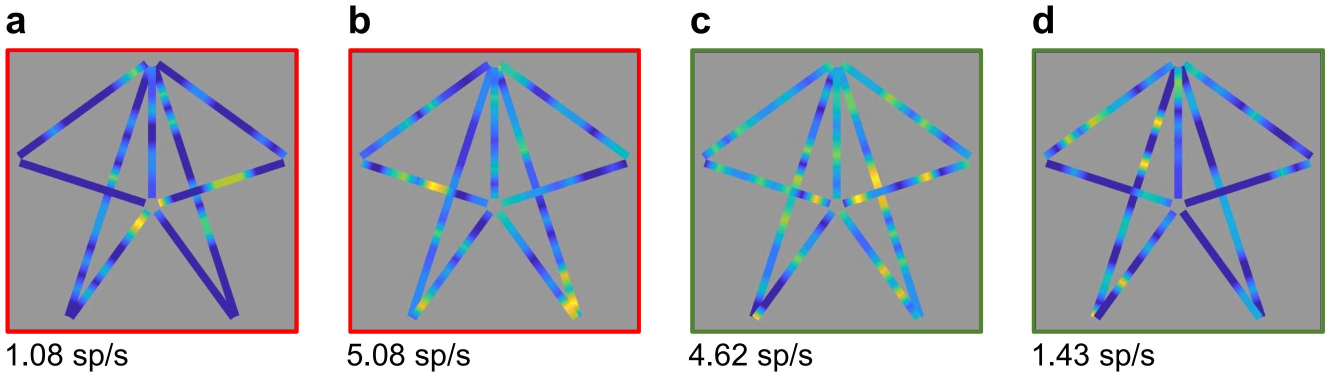

Supplementary Fig. 2 Non-spatially modulated parietal and hippocampal cells. a,b Neural place maps of PPC neurons whose activity were not modulated by monkey's position (permutation test). The color axis represents the firing rate (maximal firing rate in spike/s indicated on the bottom left of each map). For a better visualization, data are displayed from the 5th to the 99th percentiles. **c,d** Same conventions as in **a,b**, for non-spatially modulated HPC example cells.

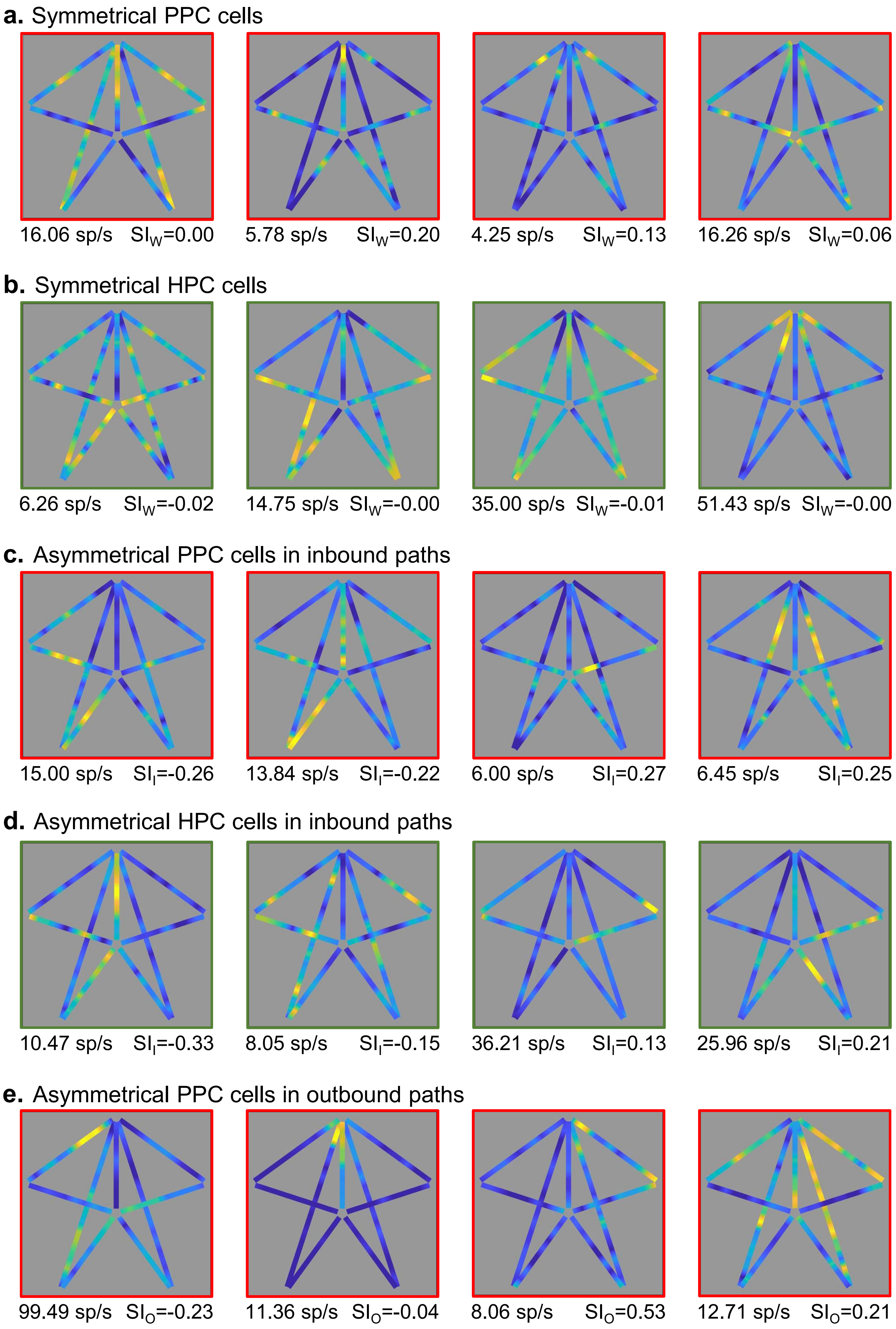

a. Symmetrical PPC cells
16.06 sp/s SI_W=0.00
5.78 sp/s SI_W=0.20
4.25 sp/s SI_W=0.13
16.26 sp/s SI_W=0.06
b. Symmetrical HPC cells
6.26 sp/s SI_W=-0.02
14.75 sp/s SI_W=-0.00
35.00 sp/s SI_W=-0.01
51.43 sp/s SI_W=-0.00
c. Asymmetrical PPC cells in inbound paths
15.00 sp/s SI_I=-0.26
13.84 sp/s SI_I=-0.22
6.00 sp/s SI_I=0.27
6.45 sp/s SI_I=0.25
d. Asymmetrical HPC cells in inbound paths
10.47 sp/s SI_I=-0.33
8.05 sp/s SI_I=-0.15
36.21 sp/s SI_I=0.13
25.96 sp/s SI_I=0.21
e. Asymmetrical PPC cells in outbound paths
99.49 sp/s SI_O=-0.23
11.36 sp/s SI_O=-0.04
8.06 sp/s SI_O=0.53
12.71 sp/s SI_O=0.21

f. Asymmetrical HPC cells in outbound paths

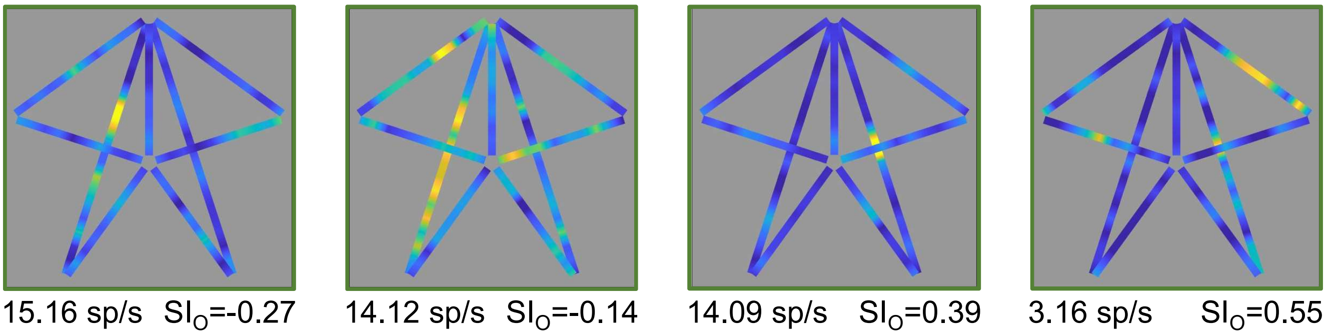

Supplementary Fig. 3 Symmetry of the parietal and hippocampal spatial activity patterns. a,b Neural place maps of example PPC (**a**) and HPC (**b**) position-modulated cells displaying a significantly symmetrical activity across the whole maze (determined by a Wilcoxon matched pairs signed-rank test; symmetry index, SI, indicated on the bottom right of each map). The color axis represents the firing rate (maximal firing rate in spike/s indicated on the bottom left of each map). For a better visualization, data are displayed from the 5th to the 99th percentiles. **c,d** Same conventions as in **a**, for PPC (**c**) and HPC (**d**) position-modulated cells displaying a significantly asymmetrical activity in the inbound paths only (determined by a Wilcoxon matched pairs signed-rank test). **e,f** Same conventions as in **a**, for PPC (**e**) and HPC (**f**) position-modulated cells displaying a significantly asymmetrical activity in the outbound paths only (determined by a Wilcoxon matched pairs signed-rank test).

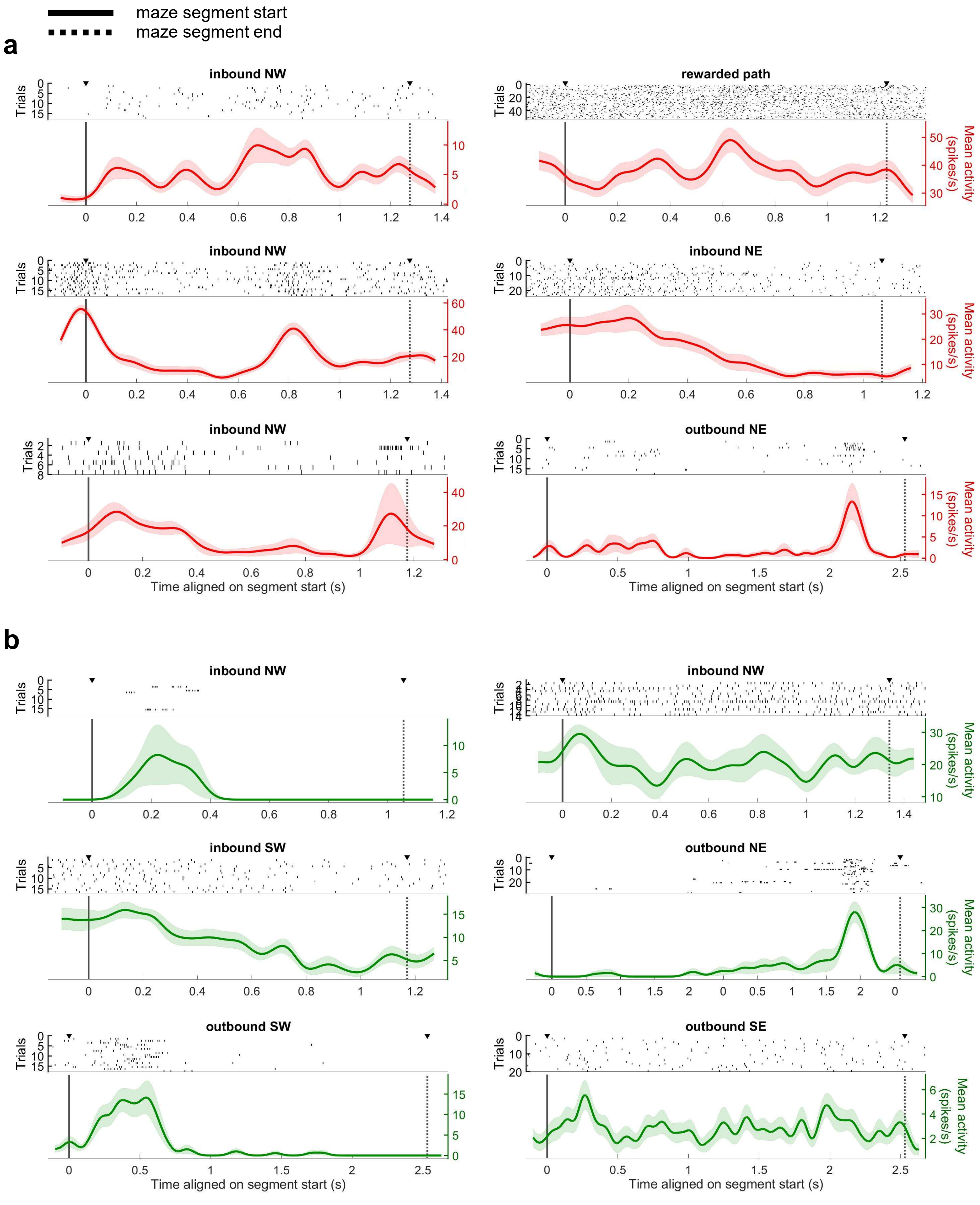

Supplementary Fig. 4 Position-related activities in parietal and hippocampal neurons. a Raster plot (top) and average activity (bottom) of each of the 6 PPC example neurons presented in **Fig. 1b-g**, aligned on their preferred segment start (black solid line). The standard errors of the mean are indicated in light color. The identity of the preferred segment is indicated on top of each plot. **b** Same conventions as for **a**, for the 6 HPC example cells, corresponding to the neurons in **Fig. 1h-m**.

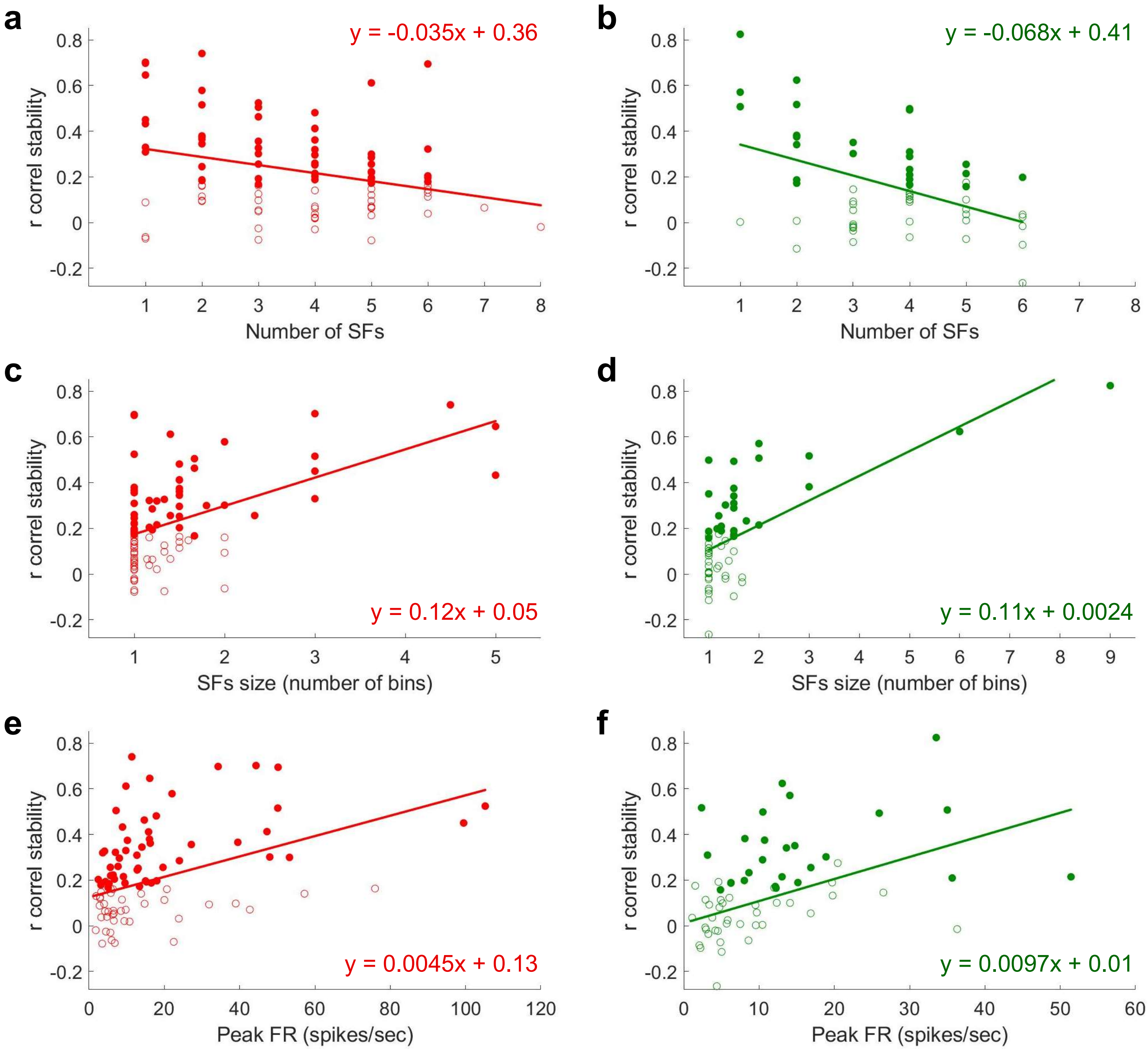
a
y = -0.035x + 0.36
r correl stability
Number of SFs
b
y = -0.068x + 0.41
r correl stability
Number of SFs
c
y = 0.12x + 0.05
r correl stability
SFs size (number of bins)
d
y = 0.11x + 0.0024
r correl stability
SFs size (number of bins)
e
y = 0.0045x + 0.13
r correl stability
Peak FR (spikes/sec)
f
y = 0.0097x + 0.01
r correl stability
Peak FR (spikes/sec)

Supplementary Fig. 5 Properties of the parietal and hippocampal spatial fields. a Stability correlation coefficient (*r*) as a function of the number of spatial fields (SFs) for the PPC spatially-modulated neurons (*N*=91). The filled markers represent cells with a significant positive correlation between the activity of the first and second halves of the session (Pearson correlation: $r \geq 0$ and permutation test: $p \leq 0.05$). The equation of the linear regression line is indicated on the top right of the plot. Source data are provided as a Source Data file. The linear regression shows a significant co-dependence between the two variables (Pearson correlation: r=-0.30, p=0.0043). **b** Same as in **a**, for the HPC spatially-modulated neurons (*N*=64). Source data are provided as a Source Data file. The linear regression shows a significant co-dependence between the two variables (Pearson correlation: r=-0.46, $p=2.44 \text{x} 10^{-4}$). **c** Same conventions as in **a**, for the stability correlation coefficient as a function of the size of the SFs (in total number of bins) of the PPC neurons (*N*=91). Source data are provided as a Source Data file. The linear regression shows a significant co-dependence between the two variables (Pearson correlation: r=0.51, $p=2.81 \text{x} 10^{-7}$). **d** Same as in **c**, for the HPC neurons (*N*=64). Source data are provided as a Source Data file. The linear regression shows a significant co-dependence between the two variables (Pearson correlation: r=0.65, $p=1.43 \text{x} 10^{-8}$). **e** Same conventions as in **a**, for the stability correlation coefficient as a function of the peak activity (in spike/s) of the PPC neurons (*N*=91). Source data are provided as a Source Data file. The linear regression shows a significant co-dependence between the two variables (Pearson correlation: r=0.38, $p=2.13 \text{x} 10^{-4}$). **f** Same as in **e**, for the HPC neurons (*N*=64). Source data are provided as a Source Data file. The linear regression shows a significant co-dependence between the two variables (Pearson correlation: r=0.41, $p=8.12 \text{x} 10^{-4}$).

a

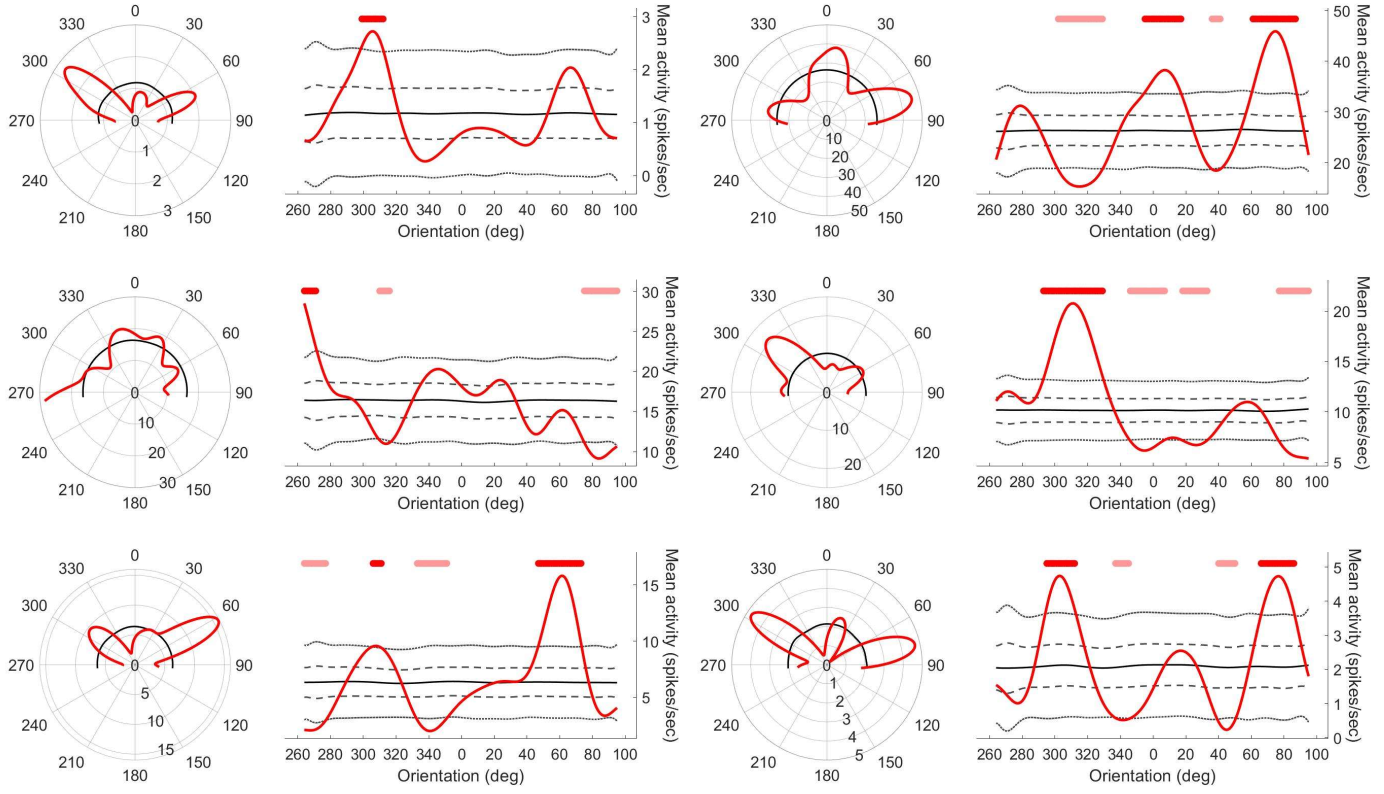

Mean activity (spikes/sec)
Orientation (deg)

b

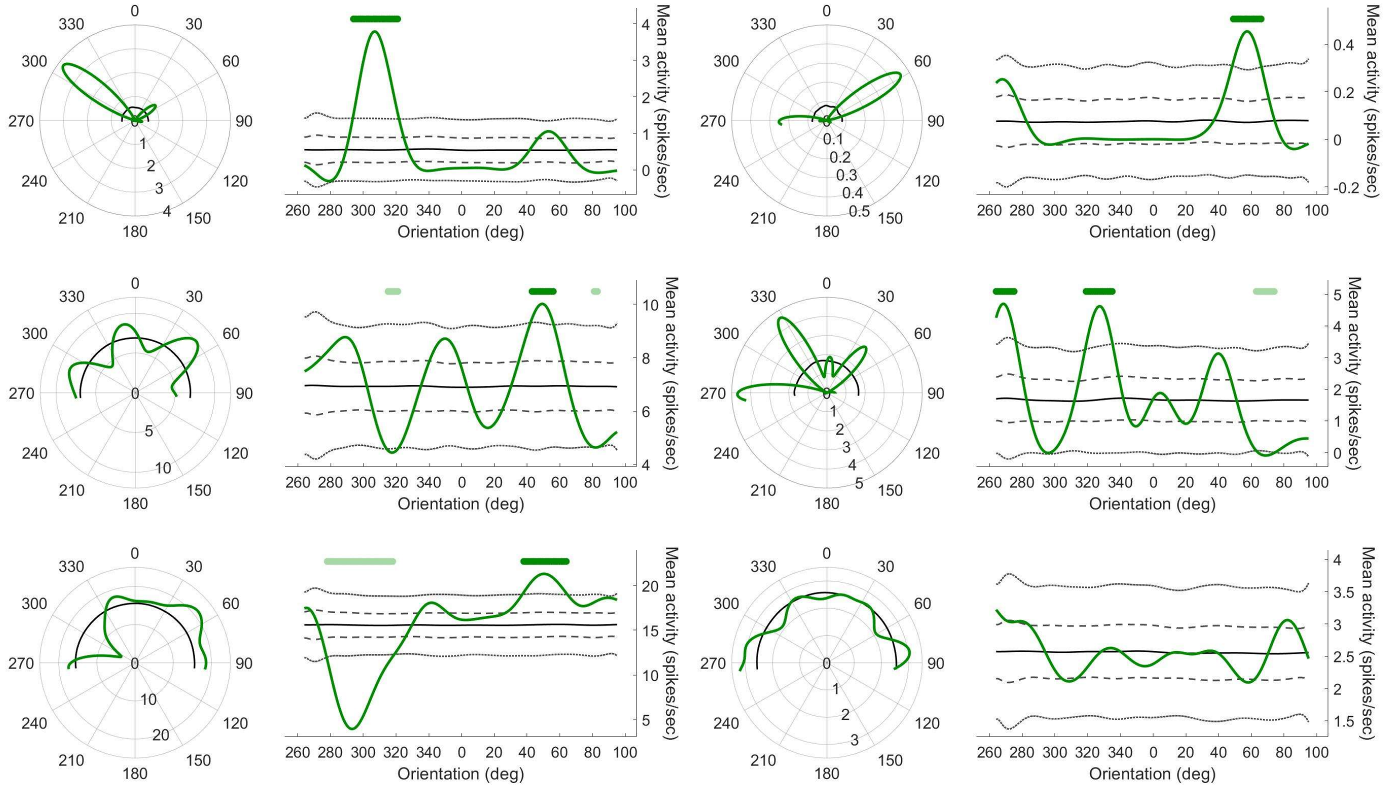

Mean activity (spikes/sec)
Orientation (deg)

Supplementary Fig. 6 Orientation-modulation in parietal and hippocampal neurons. a Polar (top) and linear (bottom) plots of the activity (in red) of the 6 PPC example neurons presented in **Fig. 1b-g**, as a function of the virtual orientation of the animal (in degrees), when located in the maze center. The North corresponds to an orientation of 0 or 360°. The South was never faced when considering only the correct trials. The lines above the linear plot indicate orientations for which the response activity was significantly higher (dark) or lower (light) than threshold (mean of surrogated data ± 2.5SD; dotted lines). The solid black line represents the mean activity of the surrogated data, and the dashed line its SD. **b** Same conventions as in **a**, for the 6 HPC example cells presented in **Fig. 1h-m**.

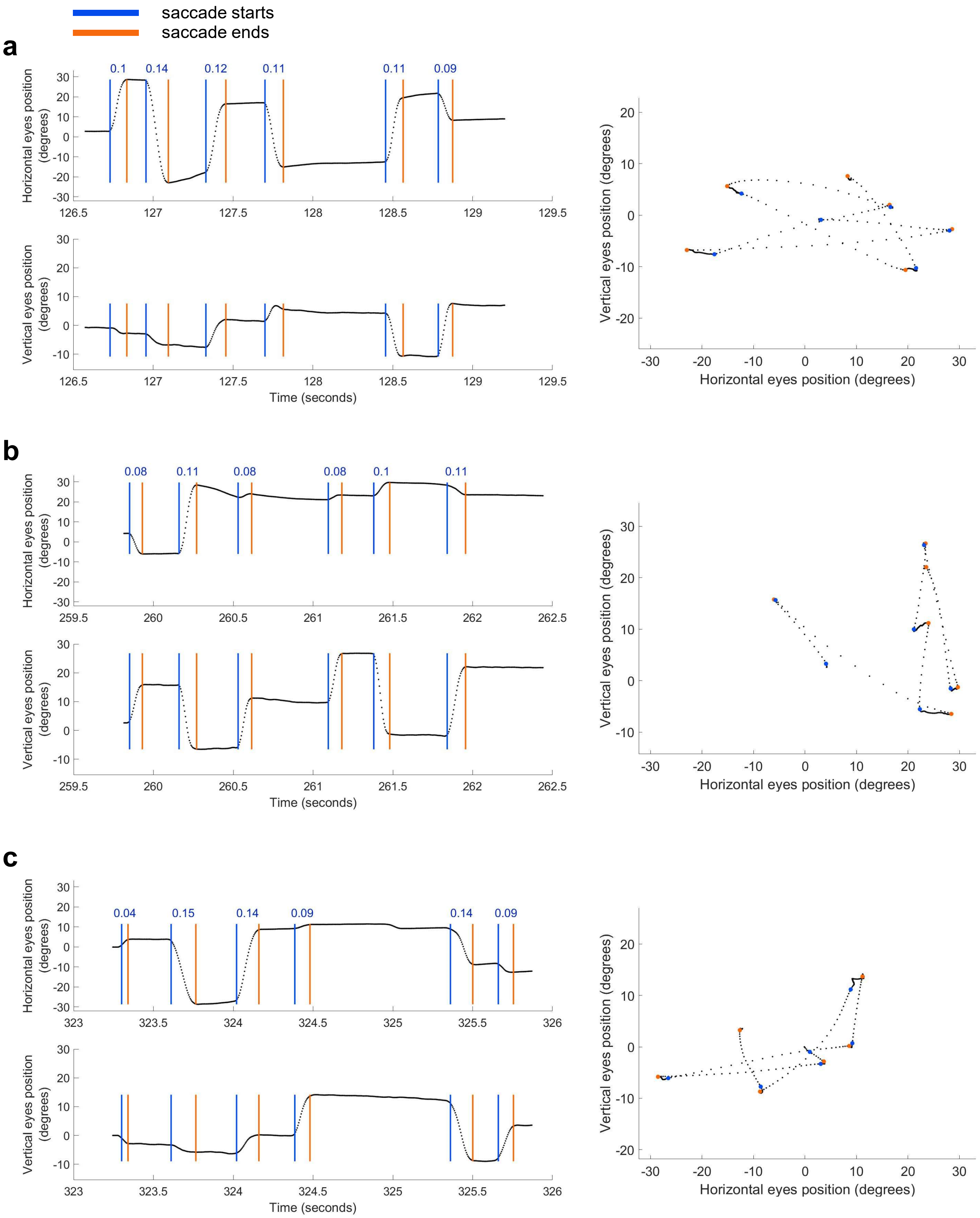

Supplementary Fig. 7 Monkeys' eye-traces. a-c Three example samples of 3-seconds horizontal (left, top) and vertical (left, bottom) eye-traces. The blue and orange vertical lines respectively mark the start and the end of the saccades. Source data are provided as a Source Data file. For each saccade, their duration (in s) is indicated on the top of the left panel, in blue. The right panels represent the same eye-traces, plotted in two dimensions.

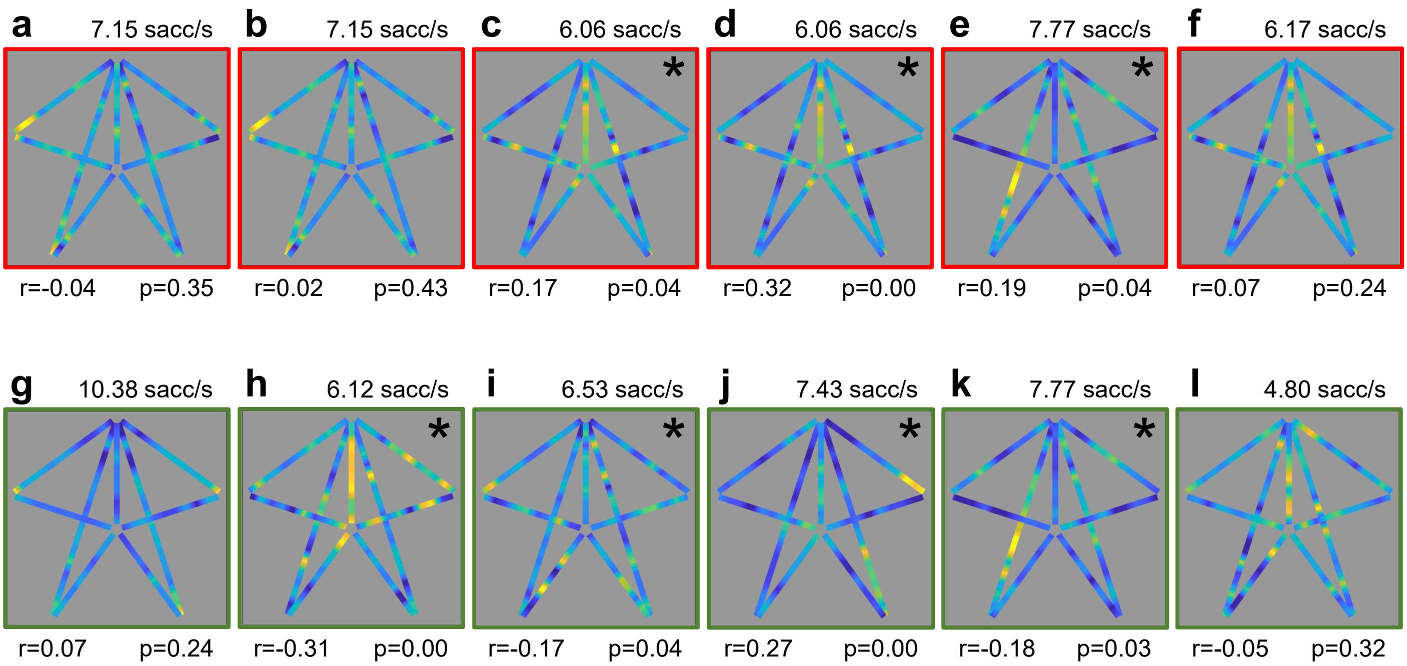

Supplementary Fig. 8 Saccades maps. a-f Maps representing the behavioral saccade rate as a function of the animal's position in the maze for example sessions during which neurons of **Fig. 1b-g** were recorded (i.e. « saccades maps »). The saccade frequency map and the neural place map (Fig. 1b-g) were significantly correlated when indicated by the asterisk (permutation tests, p-values on the bottom right of each map, and Pearson's correlation coefficient on the bottom left). For a better visualization, data are displayed from the 5th to the 99th percentiles. **g-l** Same conventions as for **a-f**, for HPC example sessions, corresponding to cells in **Fig.1h-m**.

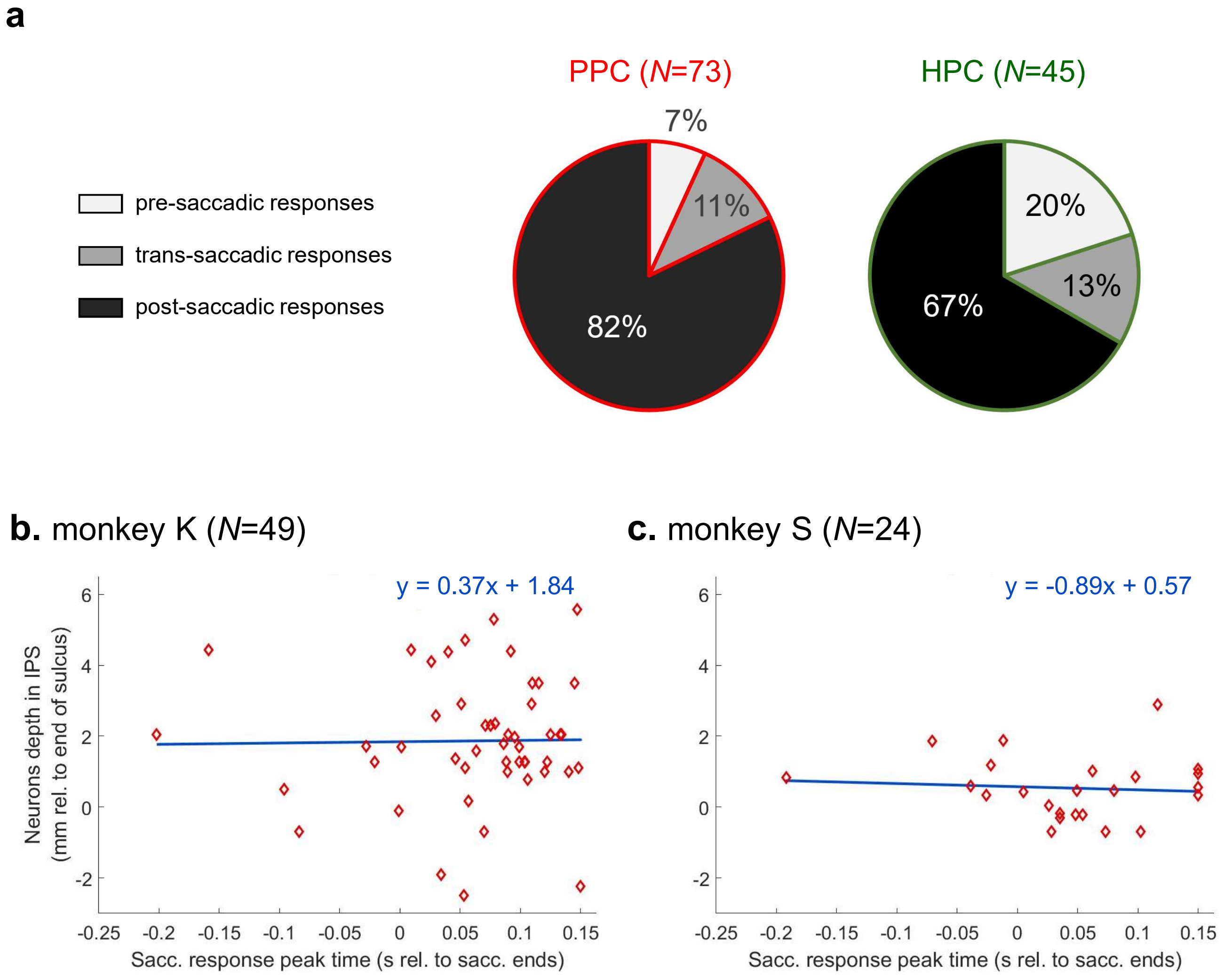

Supplementary Fig. 9 Timing of parietal and hippocampal saccade-related activities. a Proportions of neurons displaying a pre-, trans- or post-saccadic peak activity, among the PPC (N=73; in red) and HPC (N=45; in green) saccade-responsive cells. For cells presenting a mixed-response, the positive activity peaks were used for analyses. **b** Times of the peak activity of the saccade-responsive PPC neurons, relative to saccade ends, as a function of neurons' anatomical depth in the intraparietal sulcus of Monkey K ($N_{neurons}$ =49). The distance was calculated relatively to the end of the animal's sulcus. The equation of the linear regression line is indicated on the top right of the plot. Source data are provided as a Source Data file. The linear regression (in blue) shows no significant effect of the anatomical depth on the peak time (Pearson correlation: r=0.015, p=0.92). **c** Same conventions as in **b**, for Monkey S ($N_{neurons}$ =24). Source data are provided as a Source Data file. The linear regression (in blue) shows no significant effect of the anatomical depth on the peak time (Pearson correlation: r=-0.082, p=0.70).

a. PPC units with peri-saccadic response amplitude + baseline modulations

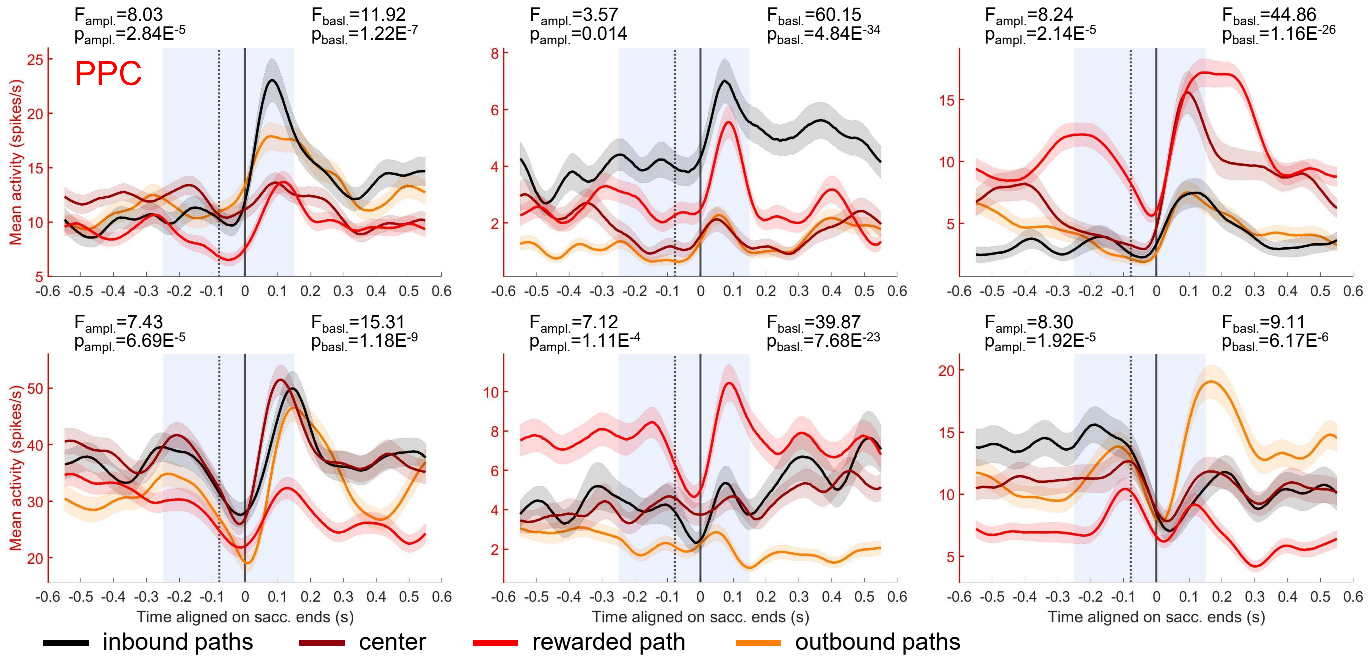

b. PPC & HPC units with only peri-saccadic baseline modulation

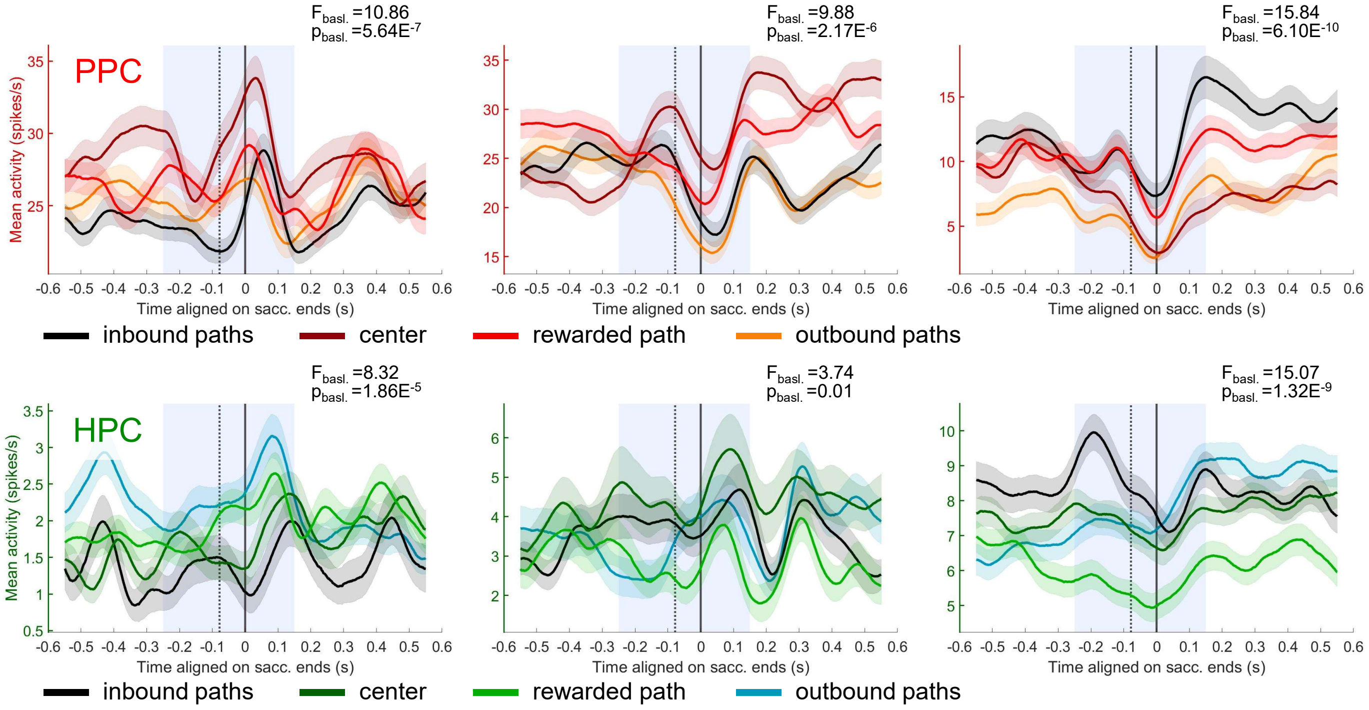

c. Proportions of preferred maze segment in cells with peri-saccadic modulation

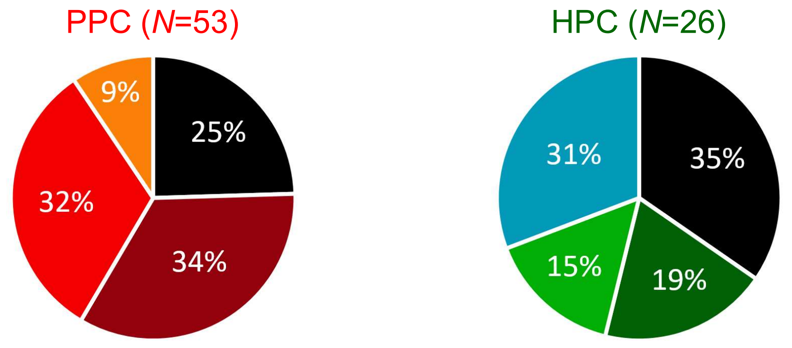

Supplementary Fig. 10 Response modulations by maze segments in the parietal and hippocampal saccade-responsive cells. a Averaged activity of example PPC cells, aligned on saccade ends (black solid line). The black dotted line represents the average time of saccade starts (-78ms). The standard errors are indicated in light color. The saccade-related activity is shown for different maze segments. All the cells displayed a significant difference of response peak amplitude (one-way-ANOVA, 3df; statistics and p-values indicated on the top left of the figures) as well as a significant difference in peri-saccadic response rate (one-way-ANOVA, 3df; statistics and p-values indicated on the top right of the figures) in the response window (in light blue). **b** Same averaged activity as in **a**, for PPC (top, in reds) and HPC (bottom, in greens) cells that displayed a significant difference in peri-saccadic response rate (one-way-ANOVA, 3df; statistics and p-values indicated on the top right of the figures), but no difference in response peak amplitude. **c** Proportions of maze segment for which the saccade-related activity was the highest, in the PPC (N=53) and HPC (N=26) cells displaying a selectivity in peri-saccadic response. In PPC, the distribution was not uniform when including the outbound paths (Chi-squared goodness-of-fit: X^2_{3df}=7.91, p=0.048), but it was in the three other segments (X^2_{3df}=0.88, p=0.65). In HPC, the distribution was uniform (X^2_{3df}=2.62, p=0.45).

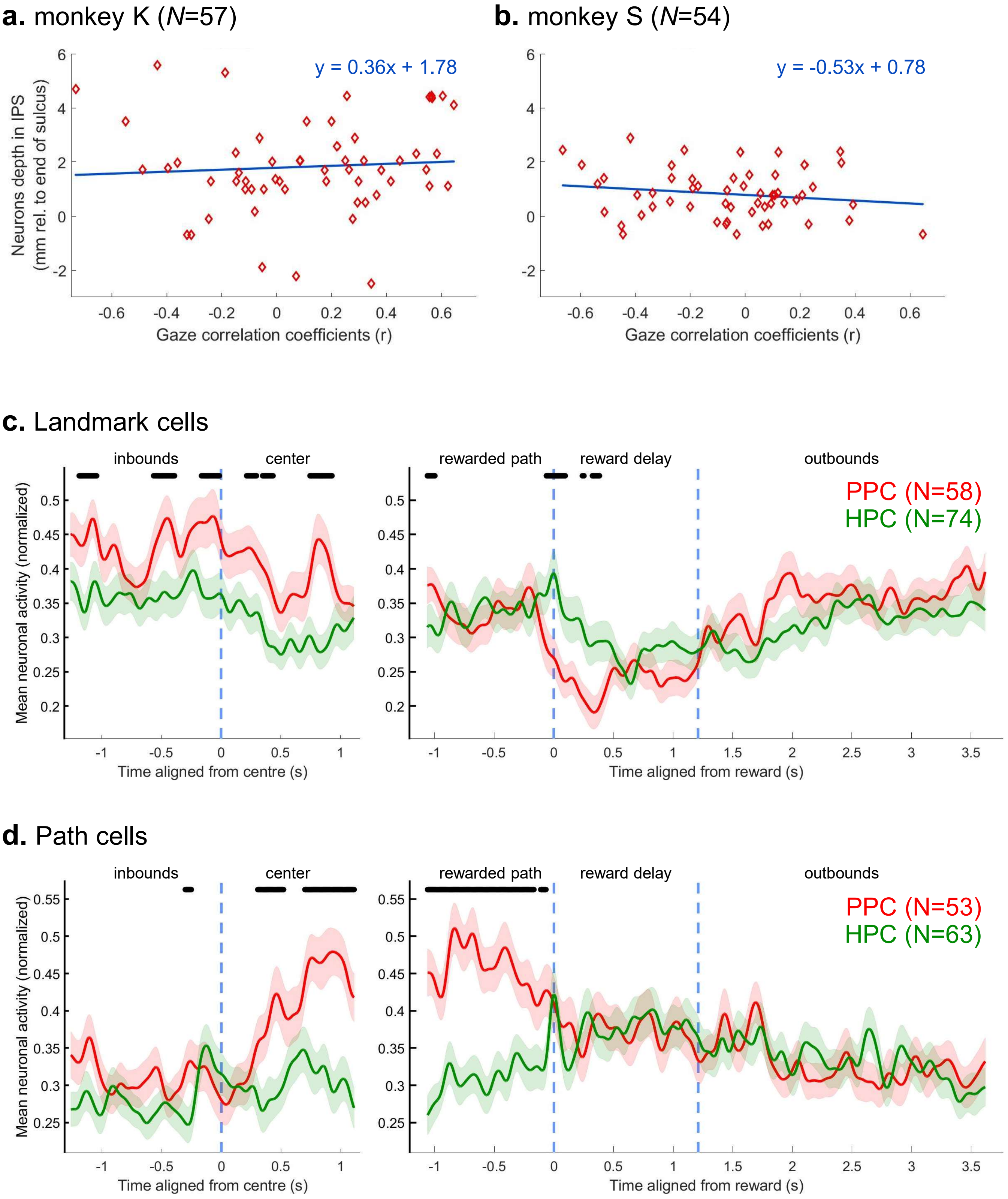
a. monkey K (N=57)
y = 0.36x + 1.78
Neurons depth in IPS (mm rel. to end of sulcus)
Gaze correlation coefficients (r)
b. monkey S (N=54)
y = -0.53x + 0.78
Gaze correlation coefficients (r)
c. Landmark cells
inbounds
center
rewarded path
reward delay
outbounds
PPC (N=58)
HPC (N=74)
Mean neuronal activity (normalized)
Time aligned from centre (s)
Time aligned from reward (s)
d. Path cells
inbounds
center
rewarded path
reward delay
outbounds
PPC (N=53)
HPC (N=63)
Mean neuronal activity (normalized)
Time aligned from centre (s)
Time aligned from reward (s)

Supplementary Fig. 11 Anatomical and spatial properties of the landmark and path cells. a Coefficients of the correlation between neurons' gaze-related activity and the reference sine-wave as a function of neurons' anatomical depth in the intraparietal sulcus of Monkey K ($N_{neurons}$=57). The distance was calculated relatively to the end of the animal's sulcus. Positive coefficients correspond to landmark cells, and negative ones to path cells. The equation of the linear regression line is indicated on the top right of the plot. Source data are provided as a Source Data file. The linear regression (in blue) shows no significant effect of the anatomical depth on the correlation coefficients (Pearson correlation: r=0.070, p=0.60). **b** Same conventions as in **a**, for Monkey S ($N_{neurons}$=54). Source data are provided as a Source Data file. The linear regression (in blue) shows no significant effect of the anatomical depth on the correlation coefficients (Pearson correlation: r=-0.16, p=0.24). **c** Averaged normalized activity of PPC (N=58, in red) and HPC (N=74, in green) landmark cells aligned on the time the monkey reached the maze center (left panel, blue dashed line) and on the reward delivery time (right panel). The second blue dashed line in right panel indicated the end of the stationary reward-delivery-delay. The black lines above indicate times for which PPC and HPC activities were significantly different (Wilcoxon rank sum). **d** Same conventions as in **c**, for the PPC (N=53) and HPC (N=63) path cells.

Fig.	IC	S	DT
1B	0.51	0.56	1.00
1C	0.17	0.77	0.89
1D	0.19	0.73	0.91
1E	0.14	0.80	0.86
1F	0.32	0.81	0.85
1G	0.51	0.40	1.00
1H	1.34	0.23	1.00
1I	0.09	0.93	0.60
1J	1.71	0.21	1.00
1K	0.08	0.90	0.88
1L	0.86	0.25	1.00
1M	0.12	0.90	0.82
S1Ca	0.97	0.28	1.00
S1Cb	0.19	0.79	1.00
S1Da	0.30	0.72	1.00
S1Db	0.73	0.40	1.00
S1La	0.12	0.85	0.82
S1Lb	0.66	0.38	1.00
S1Lc	0.56	0.52	1.00
S1Ld	0.20	0.76	0.88
S1Ma	0.17	0.83	0.87
S1Mb	0.06	0.92	0.59
S1Mc	0.08	0.90	0.88
S1Md	0.02	0.97	0.39
S1Na	0.23	0.76	0.88
S1Nb	0.05	0.92	0.63
S1Nc	0.62	0.48	1.00
S1Nd	0.41	0.63	1.00
S1Oa	0.24	0.69	1.00
S1Ob	0.14	0.84	0.90
S1Oc	0.09	0.84	0.86
S1Od	0.05	0.88	0.74
S1Pa	0.09	0.88	0.71
S1Pb	0.92	0.26	1.00
S1Pc	0.55	0.51	1.00
S1Pd	0.14	0.78	1.00
S1Qa	0.18	0.65	1.00
S1Qb	0.09	0.95	0.50
S1Qc	0.62	0.37	1.00
S1Qd	0.85	0.36	1.00

Supplementary Table 1 Spatial selectivity of PPC and HPC cells. Summary table indicating, for each cell of which the neural place map is represented in **Fig. 1** or **Supplementary Fig. 1**, their information content (IC), sparsity index (S) and depth of tuning index (DT).

		Monkey K	Monkey S	Total
Frequency (sacc/s)	inbounds	2.38±0.12	2.74±0.18	2.51±0.11
	centre	2.76±0.13	3.98±0.34	3.19±0.20
	rewarded	2.79±0.11	2.36±0.16	2.64±0.10
	outbounds	2.22±0.094	2.73±0.20	2.40±0.11
	Total	2.54±0.066	2.95±0.17	2.68±0.076
Duration (ms)	inbounds	78.49±1.58	72.70±1.49	76.45±1.31
	centre	80.86±1.81	71.22±1.64	77.46±1.69
	rewarded	75.28±2.01	72.78±0.98	74.40±1.37
	outbounds	85.06±2.80	79.19±1.80	82.99±2.02
	Total	79.92±1.16	73.97±0.96	77.83±0.89

Supplementary Table 2 Comparison of the saccade frequency and duration per monkey. Contingency table indicating the average frequency (in saccade/s) and duration (in ms) of saccades depending on the maze segments where they were performed (rows) and on the animal′s identity (columns).

	PPC (N=111)	HPC (N=137)	all areas (N=248)
landmark cells (N=132)	11.98 ± 4.27 sp/s	5.05 ± 1.38 sp/s	8.09 ± 2.11 sp/s
path cells (N=116)	7.21 ± 1.59 sp/s	4.50 ± 1.46 sp/s	5.74 ± 1.10 sp/s
all cell types (N=248)	9.70 ± 2.39 sp/s	4.73 ± 0.97 sp/s	

Supplementary Table 3 Comparison of the firing rates of PPC and HPC landmark and path cells. Contingency table indicating the peak firing rates (in spikes/sec) of the neurons depending on their « type » and their region. Landmark cells fired more than path ones (two-way-ANOVA: F_{1df} =4.72, p=0.031), parietal cells fired more than HPC ones (F_{1df} =15.5, p=1.08×10^{-4}), and the effect tended to be amplified for the parietal landmark neurons (F_{1df}=2.97, p=0.086).
