# Supplementary material for: Organizing space through saccades and fixations between primate posterior parietal cortex and hippocampus
Source: Nat Commun. 2024 Dec 1;15:10448. doi: 10.1038/s41467-024-54736-7 (PMC11609276; doi:10.1038/s41467-024-54736-7)
Supplement: Supplementary file 2 — Reporting Summary [file 41467_2024_54736_MOESM2_ESM.pdf]

nature portfolio

Corresponding author(s): Sylvia Wirth

Last updated by author(s): 03/10/2024

Reporting Summary

Nature Portfolio wishes to improve the reproducibility of the work that we publish. This form provides structure for consistency and transparency in reporting. For further information on Nature Portfolio policies, see our Editorial Policies and the Editorial Policy Checklist.

Statistics

For all statistical analyses, confirm that the following items are present in the figure legend, table legend, main text, or Methods section.

n/a	Confirmed	
☐	☒	The exact sample size (*n*) for each experimental group/condition, given as a discrete number and unit of measurement
☒	☐	A statement on whether measurements were taken from distinct samples or whether the same sample was measured repeatedly
☐	☒	The statistical test(s) used AND whether they are one- or two-sided<br>*Only common tests should be described solely by name; describe more complex techniques in the Methods section.*
☒	☐	A description of all covariates tested
☐	☒	A description of any assumptions or corrections, such as tests of normality and adjustment for multiple comparisons
☐	☒	A full description of the statistical parameters including central tendency (e.g. means) or other basic estimates (e.g. regression coefficient) AND variation (e.g. standard deviation) or associated estimates of uncertainty (e.g. confidence intervals)
☐	☒	For null hypothesis testing, the test statistic (e.g. *F*, *t*, *r*) with confidence intervals, effect sizes, degrees of freedom and *P* value noted<br>*Give P values as exact values whenever suitable.*
☒	☐	For Bayesian analysis, information on the choice of priors and Markov chain Monte Carlo settings
☒	☐	For hierarchical and complex designs, identification of the appropriate level for tests and full reporting of outcomes
☐	☒	Estimates of effect sizes (e.g. Cohen's *d*, Pearson's *r*), indicating how they were calculated

Our web collection on statistics for biologists contains articles on many of the points above.

Software and code

Policy information about availability of computer code

Data collection	AlphaLab (AlphaOmega); Plexon Offline Sorter V4; Monkey3D.
Data analysis	Custom Matlab (version R2019b) written scrips. The custom codes generating results that are deemed central to the conclusions of this study have been deposited in the OSF database, without any restrictions [https://osf.io/9jn7k/].

For manuscripts utilizing custom algorithms or software that are central to the research but not yet described in published literature, software must be made available to editors and reviewers. We strongly encourage code deposition in a community repository (e.g. GitHub). See the Nature Portfolio guidelines for submitting code & software for further information.

Data

Policy information about availability of data

All manuscripts must include a data availability statement. This statement should provide the following information, where applicable:

- Accession codes, unique identifiers, or web links for publicly available datasets
- A description of any restrictions on data availability
- For clinical datasets or third party data, please ensure that the statement adheres to our policy

The data generated in this study have been deposited in the OSF database, without any restrictions [https://osf.io/e974a/]. The processed data generated in this study are provided in the Supplementary Source Data file.

Research involving human participants, their data, or biological material

Policy information about studies with human participants or human data. See also policy information about sex, gender (identity/presentation), and sexual orientation and race, ethnicity and racism.

Reporting on sex and gender	The study does not involve human patients.
Reporting on race, ethnicity, or other socially relevant groupings	*Please specify the socially constructed or socially relevant categorization variable(s) used in your manuscript and explain why they were used. Please note that such variables should not be used as proxies for other socially constructed/relevant variables (for example, race or ethnicity should not be used as a proxy for socioeconomic status).*<br>*Provide clear definitions of the relevant terms used, how they were provided (by the participants/respondents, the researchers, or third parties), and the method(s) used to classify people into the different categories (e.g. self-report, census or administrative data, social media data, etc.)*<br>*Please provide details about how you controlled for confounding variables in your analyses.*
Population characteristics	*Describe the covariate-relevant population characteristics of the human research participants (e.g. age, genotypic information, past and current diagnosis and treatment categories). If you filled out the behavioural & social sciences study design questions and have nothing to add here, write "See above."*
Recruitment	*Describe how participants were recruited. Outline any potential self-selection bias or other biases that may be present and how these are likely to impact results.*
Ethics oversight	*Identify the organization(s) that approved the study protocol.*

Note that full information on the approval of the study protocol must also be provided in the manuscript.

Field-specific reporting

Please select the one below that is the best fit for your research. If you are not sure, read the appropriate sections before making your selection.

☒ Life sciences ☐ Behavioural & social sciences ☐ Ecological, evolutionary & environmental sciences

For a reference copy of the document with all sections, see nature.com/documents/nr-reporting-summary-flat.pdf

Life sciences study design

All studies must disclose on these points even when the disclosure is negative.

Sample size	We kept for analysis 142 hippocampal neurons and 111 parietal ones, recorded from 2 monkeys. Power calculations are independent of the number of animals and depend on the number of neurons recorded. For example, assuming a power of 0.95 at p=0.05, 150 neurons per structure will detect a 5% difference in the number of neurons coding for the variable of interest between two brain regions. Non-parametrical statistical tests were applied most of the time.
Data exclusions	Only cells whose waveforms possessed reliable signal-to-noise ratios (two-thirds of noise), high enough activity rate (activity peak superior to 1 spikes/s), and whose activity was stable in time for at least 10 trials per starting inbound paths (40 trials in total) were included in the database.
Replication	Results were obtained from a sample of neurons, and reproductivity of results were performed against surrogated data obtained from randomly shuffling the data.
Randomization	The study does not involve any group assignment for the subjects. Tests were conducted against surrogated data.
Blinding	Blinding is not applicable to our study, as our protocol did not involve specific conditions attributed to specific animal. The identity of the animal had no impact on the analyses. The analyses were applied in the exact same way for both animals and for each neuronal unit by automated scripts, without labelling having any impact on results.

Reporting for specific materials, systems and methods

We require information from authors about some types of materials, experimental systems and methods used in many studies. Here, indicate whether each material, system or method listed is relevant to your study. If you are not sure if a list item applies to your research, read the appropriate section before selecting a response.

Materials & experimental systems

n/a	Involved in the study
☒	☐ Antibodies
☒	☐ Eukaryotic cell lines
☒	☐ Palaeontology and archaeology
☐	☒ Animals and other organisms
☒	☐ Clinical data
☒	☐ Dual use research of concern
☒	☐ Plants

Methods

n/a	Involved in the study
☒	☐ ChIP-seq
☒	☐ Flow cytometry
☒	☐ MRI-based neuroimaging

Animals and other research organisms

Policy information about studies involving animals; ARRIVE guidelines recommended for reporting animal research, and Sex and Gender in Research

Laboratory animals	The study was conducted on two male rhesus macaques (Macaca mulatta), aged 5 to 6 years. Both were born in captivity (F2 generation or more), in France.
Wild animals	Study did not involve wild animals.
Reporting on sex	Sex was not implicated in our study.
Field-collected samples	Field-collected samples not implicated in our study.
Ethics oversight	All experimental procedures were approved by the animal care committee (Department of Veterinary Services, Health & Protection of Animals, permit no. 69 029 0401) and the Biology Department of the University Claude Bernard Lyon 1, in conformity with the European Community standards for the care and use of laboratory animals (European Community Council Directive No. 86–609). Further, our procedures were examined by CELYNE, the local ethics board, which approved the in vivo methods used in the laboratory.

Note that full information on the approval of the study protocol must also be provided in the manuscript.

Plants

Seed stocks	x
Novel plant genotypes	x
Authentication	x
